# Silk Fibroin/Polyvinyl Pyrrolidone Interpenetrating Polymer Network Hydrogels

**DOI:** 10.3390/polym10020153

**Published:** 2018-02-06

**Authors:** Dajiang Kuang, Feng Wu, Zhuping Yin, Tian Zhu, Tieling Xing, Subhas C. Kundu, Shenzhou Lu

**Affiliations:** 1 National Engineering Laboratory for Modern Silk, College of Textile and Clothing Engineering, Soochow University, Suzhou 215123, China; 20165215024@stu.suda.edu.cn (D.K.); 20155215019@stu.suda.edu.cn (F.W.); 20154215016@stu.suda.edu.cn (Z.Y.); 20134215013@stu.suda.edu.cn (T.Z.); xingtieling@suda.edu.cn (T.X.); 2 3Bs Research Group, Headquarters of the European Institute of Excellence on Tissue Engineering and Regenerative Medicine, University of Minho, AvePark, Barco, 4805-017 Guimaraes, Portugal; kundu@hijli.iitkgp.ernet.in

**Keywords:** silk fibroin, PVP, interpenetrating polymer network, hydrogel, protein biopolymer

## Abstract

Silk fibroin hydrogel is an ideal model as biomaterial matrix due to its excellent biocompatibility and used in the field of medical polymer materials. Nevertheless, native fibroin hydrogels show poor transparency and resilience. To settle these drawbacks, an interpenetrating network (IPN) of hydrogels are synthesized with changing ratios of silk fibroin/*N*-Vinyl-2-pyrrolidonemixtures that crosslink by H_2_O_2_ and horseradish peroxidase. Interpenetrating polymer network structure can shorten the gel time and the pure fibroin solution gel time for more than a week. This is mainly due to conformation from the random coil to the β-sheet structure changes of fibroin. Moreover, the light transmittance of IPN hydrogel can be as high as more than 97% and maintain a level of 90% within a week. The hydrogel, which mainly consists of random coil, the apertures inside can be up to 200 μm. Elastic modulus increases during the process of gelation. The gel has nearly 95% resilience under the compression of 70% eventually, which is much higher than native fibroin gel. The results suggest that the present IPN hydrogels have excellent mechanical properties and excellent transparency.

## 1. Introduction

Hydrogels are of special soft and wet material with a three-dimensional network structure and high water content, the interior presents a porous, water-dispersed system, a certain strength and soft nature. They can be prepared by physical and chemical crosslinking [1,2,3]. Due to their porous structure, good biocompatibility and mechanical properties, the hydrogels are used as cell culture, drug delivery, biosensors and tissue engineering [4,5,6].

Silk fibroin (SF), derived from *Bombyx mori* cocoons, is a widely used and studied protein polymer for biomaterial applications [7]. Silk fibroin has remarkable mechanical properties when formed into different materials, which demonstrate biocompatibility [8,9,10]. It can be processed into different kinds of materials under different conditions, mainly including solution, film, microsphere, gel, filament, nanotube and scaffold [11,12,13,14,15]. Silk hydrogels are widely used as artificial skin, drug delivery carriers, microneedle systems and, biological sensors and tissue repairing [1,13,16,17,18]. Previous studies find that the hydrogel formation and sol-gel transition of the hydrogels depend on the protein concentration, temperature and pH [19]. In order to further improve the properties of fibroin hydrogel, silk fibroin can be mixed with other various polymers or macromolecules, such as chitosan, polyvinyl alcohol, hyaluronic acid, sodium alginate, gelatin and others [16,20,21,22]. The major drawbacks of these silk fibroin hydrogels are poor mechanical strength and bad biomedical applications [1,3,19].

To overcome this drawback, in this study silk hydrogels properties are improved upon by forming interpenetrating networks of silk blended with *N*-Vinyl-2-pyrrolidone, IPN (Interpenetrating Polymer Network). The polymer composites can be fabricated by thoroughly mixing the two or more types of molecular chains [23,24]. The most important feature of IPN polymer network is the thermodynamically incompatible polymer can be mixed to form at least the kinetic stability of the composites properties of the material in order to achieve the performance between the complementary components [25]. At the same time, characteristics about IPN like special cell structure, interfacial interpenetration and biphasic continuous structural morphological make them in the performance or function to produce a special synergistic effect.

It is known that polymer of *N*-Vinyl-2-pyrrolidone (PVP) has excellent biocompatibility and inert nature. This makes them suitable for applications in medical and pharmacy, human metabolism. Besides, PVP holds outstanding hydrophilicity and lubricity, which make it suitable to composite with other hydrophobic polymer and then increase the mixing system’s hydrophilicity. Hosaka et al. mix and crosslink polyurethane with PVP monomer to produce high strength PVP hydrogel film [26,27,28,29]. Polyvinylpyrrolidone is used extensively with synthetic polymers such as polyvinyl alcohol and methyl methacrylate to prepare composite hydrogels to improve performances [30,31,32].

In this report, keeping in mind the past work and based on the excellent biosafety of hydrogel, we fabricate silk fibroin and *N*-Vinyl-2-pyrrolidone interactive network hydrogel with high mechanical strength and elasticity as well as high transparency. This may be applied to contact lens and artificial vitreous materials.

## 2. Materials and Methods

### 2.1. Materials

The fresh silk cocoons from domestic mulberry silkworm *Bombyx mori* were purchased from Suzhou Sirui Biological Technology Co., Ltd., hydrogen Peroxide 30% (Sinopharm Chemical Reagent Chemical Group Co., Ltd., Shanghai, China). Lithium bromide, *N*-vinyl-2-pyrrolidone (NVP), peroxidase from horseradish (HRP) (Aladdin Industrial Corporation, Shanghai, China). Anhydrous sodium carbonate, sodium bicarbonate, sodium chloride and all other chemicals (Sigma Chemicals Co., St. Louis, MO, USA,) used in this experiment were purchased.

### 2.2. Preparation of Regenerated Silk Fibroin

The silk cocoons (80 g) were cut into small pieces and degummed by boiling three times, in 4000 mL solution of 0.01 M Na_2_CO_3_/NaHCO_3_ in order to remove silk sericin [33]. The silk was then washed, air dried and dissolved in 9.3 M LiBr at 65 °C for 1 h followed by dialysis against deionized water for 3 days at 4 °C using cellulose dialysis membrane (MWCO 8–10 kDa) with frequent water changes. The solution was finally centrifuged at 6000 rpm for 15 min to remove impurities and precipitated matter [34].

### 2.3. Preparation of IPN Hydrogels

IPN hydrogels were prepared by free radical reaction. Briefly, silk fibroin (SF) protein concentration was adjusted to 40 mg/mL and NVP adjusted to 50 mg/mL with deionized water. Hydrogels were prepared by adding 2 mL of blended solution in centrifuge tube including SF/NVP (10:0, 9:1, 8:2, 7:3, 6:4, 5:5), 1 mM H_2_O_2_ and 10 unit/mL HRP. To this mixture, SF and NVP were added according to the blending ratios (Table 1). The solutions were allowed to gel in an incubator at 37 °C. The samples were finally removed from the mold, washed and immersed in deionized water for 24 h to remove unreacted monomers.

### 2.4. Morphology of PVP-SF IPN Hydrogels

To characterize the internal structure of IPN, the hydrogel samples were observed using a scanning electron microscope (Hitachi TM3030, Hitachi Ltd., Tokyo, Japan). The samples were frozen by liquid nitrogen and then the sample were placed in a freezing dryer (Christ) and dried for 24 h to remove water. SEM images were acquired after gold sputtering at operating voltage of 15 kV [35].

### 2.5. FTIR Measurement

All infrared spectra were recorded in the range of 600–1800 cm^−1^ using Thermo Nicolet 570 FTIR spectrometer (Nicolet Co., Madison, WI, USA). Each spectrum was acquired by accumulation of 16 scans with a resolution of 4 cm. Background measurements were subtracted from sample readings.

### 2.6. Wide-Angle X-ray Diffraction Measurement

The crystalline state of Freezing-dried IPN hydrogels was tested by XRD. Test conditions: tube voltage 40 kV, tube current 35 mA, scanning rate of 2°/min and using Cu Kα rays. XRD patterns were recorded in the 2θ region from 10° to 40°.

### 2.7. Light Transmittance Characterization

SF pure hydrogels and IPN hydrogels were equilibrated in 24-well plates and equilibrated in a 37 °C incubator for 24 h. The transmittance values were measured at 492, 550, 700 nm using a multifunctional microplate reader [36]. The thickness of the hydrogel in the experiment is controlled below 5 mm and the temperature is maintained at 37 °C.

### 2.8. Mechanical Properties

#### 2.8.1. The Compressive Stress—Strain Curve of IPN Gels

In order to compare the compressive properties of hydrogels with different mass ratios, SF pure hydrogels and IPN hydrogels with different ratios were prepared. Cylindrical hydrogel samples with flat and parallel surfaces were prepared and the initial diameter and height of samples were 10 ± 0.5 mm and 8 ± 0.5 mm respectively. Compressive strength was determined by using Universal Testing Machine (Instron-3365, Instron Co., Norwood, MA, USA) at 25° and 65% relative humidity. The test speed was set at 10 mm/min. All data were collected six samples (*n* = 6). 

#### 2.8.2. Resilience Test of IPN Gels

SF pure hydrogel and IPN hydrogel with different ratios were made into cylinders with a diameter of 10 ± 0.5 mm and a height of 8 ± 0.5 mm. The samples were compressed twice and the compression displacement was 70% of the original height of the samples. The variation curve was analyzed in the system software and the parameter values are obtained and the variation law was analyzed [37]. Test conditions: control the lifting arm speed of 1 mm/s, the trigger force of 10 g, the test used in the circular probe model P/2, detection accuracy ≥0.015%.

### 2.9. Enzymatic Degradation of IPN Gels In Vitro

IPN hydrogels were immersed in PBS (phosphate buffer saline) (37 °C, pH: 7.4) containing protease XIV (2 U/mL, Sigma-Aldrich, St. Louis, MO, USA) at a bath ratio of 1/100 (*w*/*v*). Hydrogels peptide content derived from enzymatic degradation was measured by sampling at designated time points, 4 parallel samples each group [38]. Percentage enzymatic IPN hydrogels degradation was calculated using Equation (1) mentioned above.

(1) $$\mathrm{WeightRemain}\%=\frac{m_{2}}{m_{1}}\times 100\%$$

where *m*_1_ and *m*_2_ are the mass of hydrogels before and after immersion in PBS.

## 3. Results 

### 3.1. Preparation of IPN Hydrogels

**Scheme 1:**
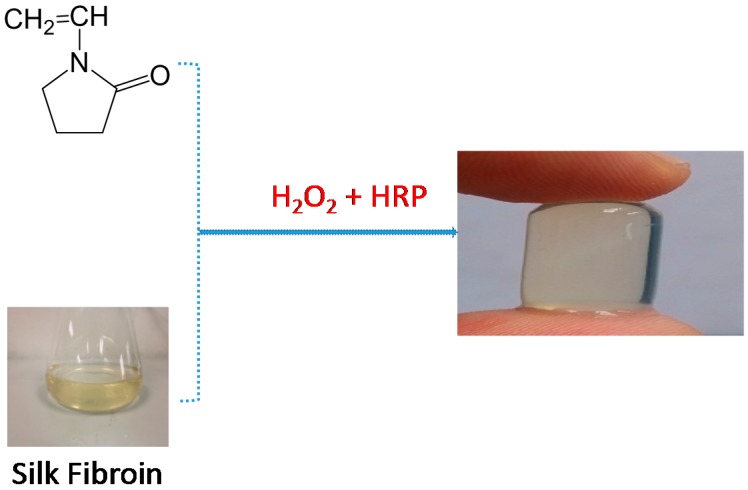
Formation of PVP–SF IPN hydrogel using HRP-H_2_O_2_ system.

Interpenetrating network hydrogel were fabricated mixing solutions of silk fibroin with NVP solutions, HRP and H_2_O_2_. During this process, NVP was used to generate free radicals under the catalysis of initiator H_2_O_2_ and horseradish peroxidase (HRP), which initiated the polymerization of monomers to produce polyvinylpyrrolidone and reacted with silk fibroin macromolecule entanglement, ultimately formed the interpenetrating network (IPN) hydrogel (Scheme 1).

### 3.2. Morphology of IPN Hydrogels

**Figure 1:**
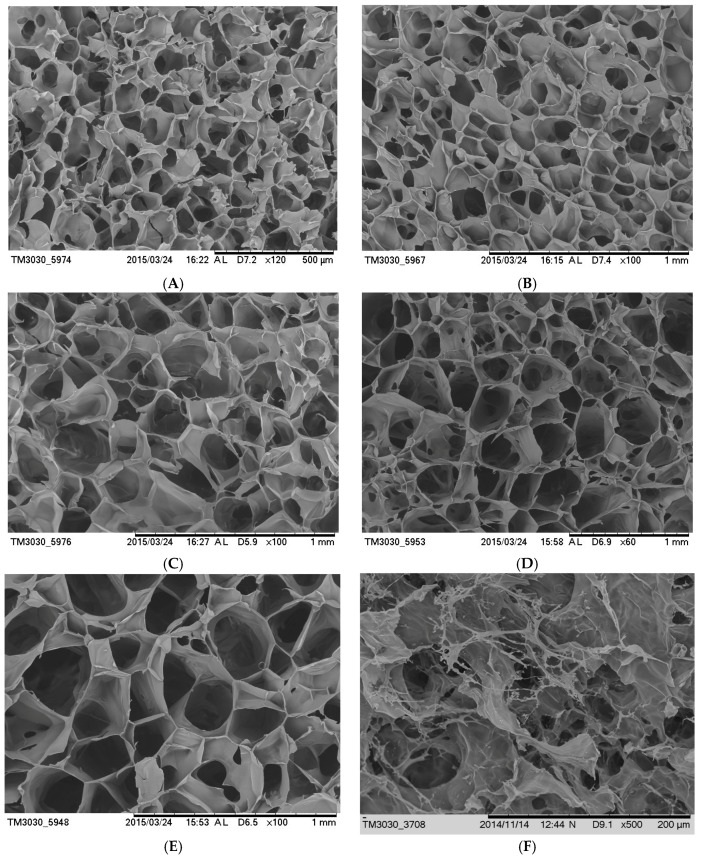
SEM images of cross-section morphology with different ratios of SF/NVP: (**A**) 90/10, (**B**) 80/20, (**C**) 70/30, (**D**) 60/40, (**E**) 50/50 and (**F**) pure SF hydrogel.

The IPN hydrogels performed dense and uniform porous structures (SEM, Figure 1). The SEM images also revealed that regular pore structure with clear pores and high porosity within PVP-SF IPN hydrogels. The pore sizes ranged from 80 to 200 μm, which were much larger than the internal pore sizes of pure silk hydrogels. The larger pores allow for the efficient delivery of the water and nutrients inside the gel. This provides sufficient space and suitable environment for cell growth and proliferation. Decrease in silk fibroin content, the internal porosity of the hydrogel became larger and uniform. With the increase of NVP content, the conversion rate decreased. This would reduce the entanglement points of polymer and silk fibroin macromolecules. This results in the internal structure of the hydrogel loose and the pores became larger.

### 3.3. Conformation and Aggregation Structure of Fibroin Hydrogels

**Figure 2:**
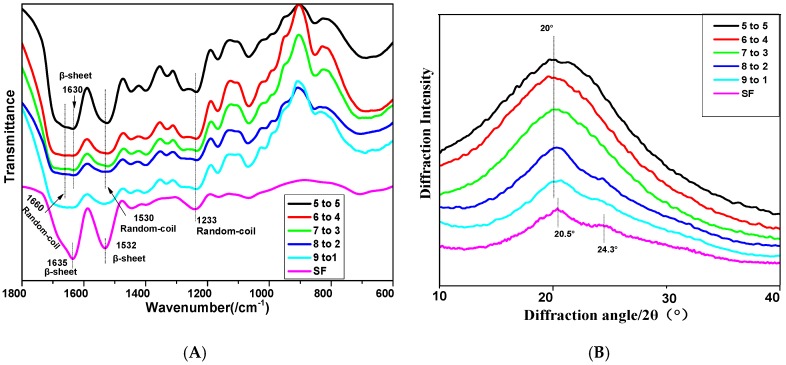
Structure of fibroin hydrogels. (**A**) FTIR; (**B**) X-ray diffraction. The solid content of hydrogel was 10 g/L.

The FITR spectra of the freeze-dried IPN hydrogel and pure SF hydrogel are shown in Figure 2. There was no significant difference between different proportions of interpenetrating network hydrogels of the FITR spectra (Figure 2A). The pure SF hydrogel exhibits characteristic absorption peaks at 1233, 1532, 1635 cm^−1^ which are the characteristic peaks of the β-sheet structure [39]. SF/PVP IPN hydrogels had obvious absorption peaks at 1530, 1630 cm^−1^ which are the characteristic peaks of random coil of SF [39]. The results indicate that the main structure of SF in IPN hydrogels is random coil.

Figure 2B shows the XRD diffraction curve of IPN hydrogel material. X-ray diffraction is an effective method for studying crystal materials and amorphous material structures [40]. There was no significant difference between the XRD diffraction curve of different proportions of interpenetrating network hydrogels (Figure 2B). It can be observed from the figure that the IPN hydrogel had a large bunted peak at 20°, which was characterized by a typical amorphous structure. That was consistent with the FTIR spectrum. Whereas the pure silk gel at 20.3° and 24.3° with a sharp absorption peak. This indicated that the pure silk fibroin had a certain crystal structure (Figure 2B).

### 3.4. Compression Mechanical Properties

**Figure 3:**
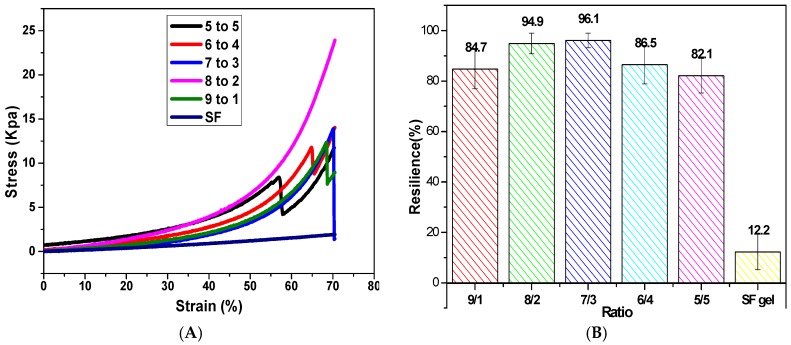
Compression mechanical properties. (**A**) Stress-strain curves of PVP-SF IPN hydrogels. (**B**) Stress resilience of PVP-SF IPN hydrogels. The solid content was 10 g/L, the temperature was 25 °C and the relative humidity was 65%.

In the stress-strain curve, both the hydrogel samples showed some nonlinear behaviors. With the increase of strain, the compressive stress on the material increased and the pure silk fibroin hydrogel appeared at about 50% compression compressive rupture phenomenon (Figure 3A). The IPN hydrogel material in the maximum compression up to 70% of the stress was still increased. The material remained intact and the material quickly rebound after the pressure was removed. The results showed that the internal structures of IPN hydrogel were regular, cross-linked closely, excellent compressive strength and resilience. IPN hydrogel significantly enhanced the compressive strength and compressive resilience of the hydrogel because of its macromolecule network entanglement.

The sample of IPN hydrogel was compressed by TPA (Test process analysis) test for consecutive compressions. Resilience is the ability of the sample to rebound during the first compression, which is the ratio of the elastic energy released by the sample upon return during the first compression cycle to the energy dissipated by the probe during compression. The sample of IPN hydrogel was compressed by TPA test for two consecutive compressions. IPN hydrogel materials have a high rebound rate of up to 95% resilience, compared with only about 12% of pure silk fibroin hydrogel. Due to the network interpenetrating structure within the material, the entanglement and the physical crosslink density of the molecular chain were greatly increased. The material underwent elastic deformation when being compressed without any damaged. A texture analyzer was used to measure and evaluated the mechanical differences between different ratios of PVP-SF IPN hydrogel and pure silk hydrogel. Notably, both the gel samples showed non-linear behavior. Stress of the hydrogel showed an increasing trend with an increase in strain. The compression fracture occurred when the pure silk gel was compressed by about 50%. IPN hydrogel at the maximum compression up to 70% of the stress was still increased. The shape was complete and the rapid removal of hydrogel after the pressure rebound, the IPN hydrogel showed higher mechanical properties compared with the pure silk hydrogel. This significantly increased the compressive strength and compressive resilience of the hydrogel by virtue of the regular cross-linking of the internal structure of the macromolecule network. This is summarized in Figure 3A,B.

### 3.5. Light Transmittance Characterization

The contact lens material of contact optical system consists, corneal related part, contact lens and the formation of tears in the cornea [41]. The copolymer hydrogels are used in the application of corneal contact lens materials, which is required to have good light transmission. When light propagates through a polymeric material, it causes a material-valence electron transition that converts part of the light energy into heat energy. This causes the light to decay. Based on the thickness of the artificial cornea and contact lens, the actual thickness of the hydrogel (4.4 mm) was corrected to the light transmittance.

**Figure 4:**
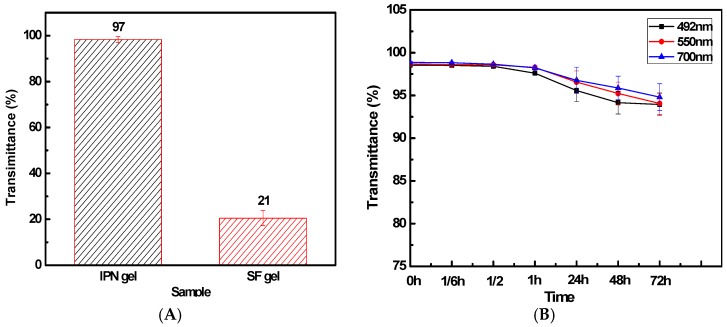
Light transmittance (**A**) at 550 nm. (**B**) IPN hydrogel transmittance at different times; The solid content of IPN hydrogel was 10 g/L, SF:NVP was 8:2 and the thickness of material was 4.4 mm.

IPN hydrogels formed for 24 h and the transmissivity reached more than 97%. This is more than national standard. The unstinted soft contact lens transmission ratio is greater than 92% [42]. The transmittance of pure silk hydrogel is only about 21% (Figure 4A,B). IPN hydrogel showed good light transmission properties. The results indicate that the structure of the hydrogel material chain is interpenetrating network structure and the pure silk hydrogel substantially crystalline structure. This hinders the light pass through, so the light transmittance is low. IPN hydrogels still retain good light transmission over time. The light transmission remains as high as 80% over a week. This is mainly due to the high degree of entanglement of the molecular chains endow the hydrogel stable internal non-crystalline structure. This insures its excellent light transmission properties.

### 3.6. Enzymatic Degradation of Hydrogels In Vitro

**Figure 5:**
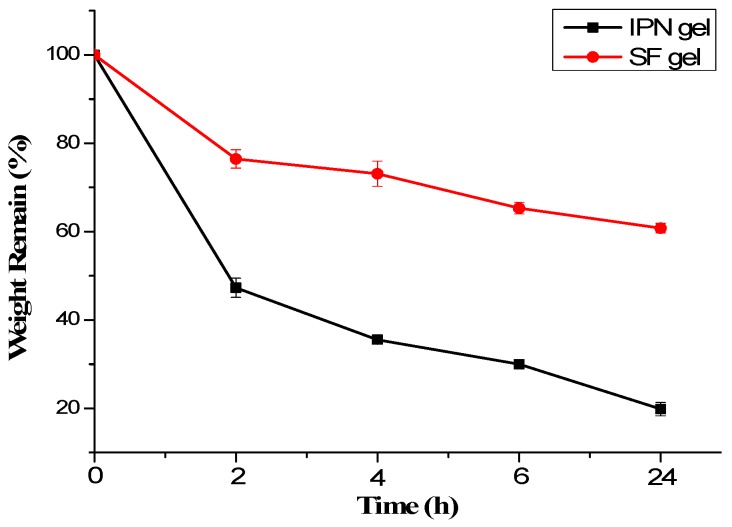
Degradation of silk fibroin hydrogels. In 2 U/mL protease XIV PBS solution (37 °C, pH: 7.4). The solid content of SF hydrogel was 10 g/L, SF:NVP was 8:2 and the thickness of material was 4.4 mm.

Interpenetrating network structure in the loss of a macromolecule, the degree of crosslinking decreased rapidly dissolved in water. The rate of degradation of the hydrogel was up to over 80% in 24 h, whereas pure silk fibroin hydrogel was degraded only about 40% (Figure 5). The main reason might be due to the enzymatic action, the silk fibroin in the IPN gel was degraded. This resulted in a decrease in the degree of crosslinking of the gel and rapid degradation. Thus, the IPN gel was a cross-linked network of hydrogels formed by intertwining and cross-linking of silk fibroin molecules and PVP molecules.

## 4. Discussion

Reports over the past decades have stated that silk hydrogel was prepared by ultrasonic induction, temperature, pH or crosslinking agent [21,43,44,45]. The silk hydrogel prepared by these methods, which exhibit a weak resilience and low light transmission, because of β-sheet structure. Meanwhile the toxicity of chemical crosslinking agents was present, which may limit its practical use. To improve the resilience and light transmission as well as the biosecurity, we prepared PVP-SF IPN hydrogel using HRP-H_2_O_2_ systems.

**Scheme 2:**
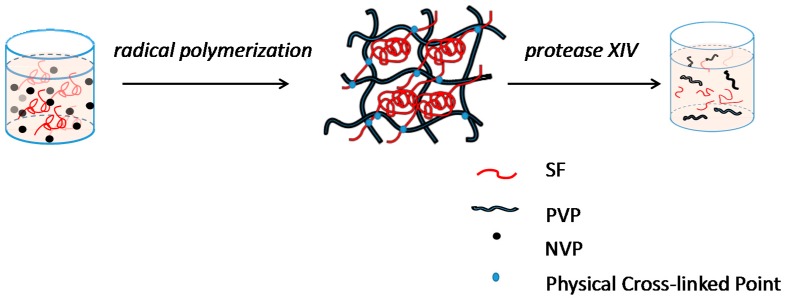
Formation and degradation of PVP–SF IPN hydrogel.

As shown in Scheme 2, the SF molecules form a loose globular structure in the solution with random coil structure. When NVP added into SF solution, the small NVP molecules disperse in water and in loose globular formed by SF macromolecules. When PVP macromolecules synthesized by the free radical polymerization of NVP in HRP-H_2_O_2_ systems, the polymerization occurs in the solvent (water), as well the interface of SF macromolecules and water. Then the crosslinking points of physical entanglement are formed by segments of SF macromolecules and segments of PVP macromolecules, which can be called interpenetrating network. When the crosslinking density rises to the critical value, the viscosity of the solution rises to infinity and the IPN hydrogel is formed. The movement of silk fibroin chain is limited due to the existence of physical crosslinking. Thus, the silk fibroin chain segment is difficult to rotate freely to form a stable β-sheet structure. That is why the structure of SF in IPN hydrogel is mainly random coil, while the structure of pure SF hydrogel is mainly β-sheet (Figure 2A).

The formation of larger particles or silk II crystals, which may make hydrogel opaque, is also limited by the interpenetrating network structure formed by SF and PVP. That is the reason why PVP-SF IPN hydrogel has good transparency and can remain for a long time. The transmittance of PVP-SF IPN hydrogel (8:2) is 97% significantly higher than that of pure silk fibroin hydrogel (21%) (Figure 3). 

When PVP-SF IPN hydrogel was immersed into protease XIV PBS solution, the chain of SF molecular was cut into small pieces by the enzyme while the chain of PVP will not be cut and still keep long chain. However, due to SF molecular was cut from long chain to small fragments, the crosslinking point of physical entanglement will be destroyed. And then the interpenetrating network structure was break up. The PVP and small fragments of SF was dissolved in water. The degradation process is very fast due to the structure of SF is random coil but not β-sheet and It is no need to completely degrade SF molecular while only need cut the point of physical entanglement. That is why the degradation rate of PVP-SF IPN hydrogel is so far faster than that of pure silk hydrogel (Figure 5).

In summary, the PVP-SF IPN hydrogels fabricated are superior to the pure silk fibroin hydrogel in terms of mechanical properties, light transmittance and biodegradability due to the mutual network structure.

## 5. Conclusions

We prepared a series of PVP–SF IPN hydrogels with high desirable and tunable features for biomedical applications. The PVP–SF IPN hydrogels have an excellent translucency (97%) degree, higher transmittance, higher compressive strength and compressive resilience and rapid protease degradation as compared to pure SF hydrogels. We also demonstrated that, by varying the concentration of silk fibroin in the IPN hydrogel, the structural, pore size and Compression mechanical properties of the resulting IPN hydrogels could be tuned. It is expected that this type of hydrogel can be used in cell migration investigation and also where transparency films are needed as biomedical materials.

## Figures and Tables

**Table 1:** Indicates the content of each component in the mixed system.

Composition	40 mg/mL SF (μL)	50 mg/mL NVP (μL)	Deionized Water (μL)
SF/NVP: 10/0	500	0	1298.6.6
SF/NVP: 9/1	450	40	1308.6
SF/NVP: 8/2	400	80	1318.6
SF/NVP: 7/3	350	120	1328.6
SF/NVP: 6/4	300	160	1338.6
SF/NVP: 5/5	250	200	1348.6

All components were added H_2_O_2_ 68 μL (1 mg/mL), HRP133.4 μL (0.25 mg/mL). The final volume of the mixed solution was 2 mL. The solid content was 10 mg/mL, the temperature was 25 °C and the relative humidity was 65%.
